# Gut microbiota dysbiosis as an inflammaging condition that regulates obesity-related retinopathy and nephropathy

**DOI:** 10.3389/fmicb.2022.1040846

**Published:** 2022-11-02

**Authors:** Jie Li, Jun-lin Lv, Xin-yue Cao, Hai-ping Zhang, Yu-jun Tan, Ting Chu, Li-li Zhao, Zhong Liu, Yu-shan Ren

**Affiliations:** ^1^Yantai Key Laboratory of Pharmacology of Traditional Chinese Medicine in Tumor Metabolism, Binzhou Medical University, Yantai, China; ^2^Endocrine and Metabolic Diseases Hospital of Shandong First Medical University, Jinan, China; ^3^School of Life Sciences, Jiangsu Normal University, Xuzhou, China; ^4^State Key Laboratory of Generic Manufacture Technology of Chinese Traditional Medicine, Lunan Pharmaceutical Group Co., Ltd., Linyi, China

**Keywords:** gut microbiota (GM), inflammaging, obesity, diabetic nephropathy, diabetic retinopathy

## Abstract

Diabetes-specific microvascular disease is a leading cause of blindness, renal failure and nerve damage. Epidemiological data demonstrated that the high morbidity of T2DM occurs as a result of obesity and gradually develops into serious complications. To date, the mechanisms that underlie this observation are still ill-defined. In view of the effect of obesity on the gut microflora, Lepr^db/db^ mice underwent antibiotic treatment and microbiota transplants to modify the gut microbiome to investigate whether microbes are involved in the development of diabetic nephropathy (DN) and/or diabetic retinopathy (DR). The mouse feces were collected for bacterial 16S ribosomal RNA gene sequencing. Cytokines including TNF-α, TGF-β1, IFN-γ, IL-1β, IL-6, IL-17A, IL-10, and VEGFA were detected by enzyme-linked immunosorbent assay (ELISA), flow cytometry, real-time PCR and immunofluorescent assay. Eyes and kidney were collected for histopathological assay. Intestinal permeability was also detected using Evans Blue. The results showed that obesity influenced metabolic variables (including fast/fed glucose, insulin, and triglyceride), retinopathy and nephropathy, and the gut microbiota. Obesity mainly reduced the ratio of Bacteroidetes/Firmicutes and influenced relative abundance of Proteobacteria, Actinobacteria, and Spirochetes. Obesity also increased intestinal permeability, metabolic endotoxemia, cytokines, and VEGFA. Microbiota transplants confirm that obesity aggravates retinopathy and nephropathy through the gut microbiota. These findings suggest that obesity exacerbates retinopathy and nephropathy by inducing gut microbiota dysbiosis, which further enhanced intestinal permeability and chronic low-grade inflammation.

## Introduction

As a metabolic disorder disease with a high incidence, type 2 diabetes mellitus (T2DM), which is characterized by high blood sugar concentration, insulin resistance, and insulin insufficiency (National Institute of Diabetes and Digestive and Kidney Diseases, 2016), affected more than 405.6 million people in 2018, and this number is estimated to increase to 510.8 million worldwide in 2030 (Basu et al., 2019). The high morbidity of T2DM occurs as a result of obesity, lack of exercise and high genetic risk (World Health Organization, 2011), and serious complications can occur as a result of the prolonged course of T2DM, which is associated with a 10-year reduction in life expectancy (Melmed et al., 2015). Among the complications of diabetes, micro- and/or macrovascular complications such as diabetic nephropathy (DN) and diabetic retinopathy (DR) are prevalent complications that occur within 5 years and are associated with high mortality of T2DM (Dunkler et al., 2015), resulting in substantial economic and social burdens (International Diabetes Federation, 2017). According to an epidemiological investigation from the World Health Organization (WHO), approximately 10 and 2% of diabetic people experience visual impairment and blindness, respectively, and these rates are predicted to double by the year 2030 (World Health Organization, 2011). In addition, end-stage renal disease (ESRD) is primarily caused by DN and affects approximately 35% of all diabetic patients worldwide (Rask-Madsen and King, 2013). As progressive diseases and T2DM-induced complications, the pathogenesis of both DR and DN is extremely complex because of multiple factors, including many different cells, molecules, and factors.

Obesity is considered a primary initiator of negative health effects because it is associated with low-grade chronic inflammation, which makes individuals prone to insulin resistance and T2DM (Olefsky and Glass, 2010). A few decades ago, the hypothesis that obesity affects the immune system, which subsequently contributes to the development of T2DM, was proposed, and this hypothesis was confirmed by subsequent research (Saltiel and Olefsky, 2017). On the one hand, the circulating levels of tumor necrosis factor α (TNF-α), interleukin-1 (IL-1), and IL-6 were significantly increased and resulted in T2DM in obese humans and high-fat diet (HFD)-induced obese mice (Luck et al., 2015; Zhou et al., 2018; Scheithauer et al., 2020). On the other hand, obesity-induced fatty accumulation in tissues such as liver, muscle, pancreas, brain, and adipose tissue imbalances the immune system, including elevated proinflammatory immune cells (M1 macrophages, CD8^+^ T cells, Th1 T cells, B cells, and natural killer (NK) cells) and reduced anti-inflammatory immune cells [M2 macrophages, regulatory T cells (Tregs), and type 2 innate lymphoid cells (ILC2s)], which ultimately results in the development of T2DM (Schwartz et al., 2016; Winer et al., 2016; Reilly and Saltiel, 2017; Painter and Akbari, 2021).

Recent studies found that the microbiota harbored in the gastrointestinal tract plays a pivotal role in the maintenance of organismal homeostasis and stable physiology (Levy et al., 2016). Dysbiosis of the intestinal microbiota is considered to play a vital role in fostering the progression of obesity-associated T2DM and diabetic complications (Garidou et al., 2015; Fernandes et al., 2019). Previous studies have demonstrated that an increase in the ratio of *Firmicutes* to *Bacteroidetes* in the gut microbiota is associated with obesity and T2DM (Cani et al., 2008). In addition, dysbiosis of the gut microbiota might modulate the immune system and ultimately induce chronic low-grade inflammation, which is already recognized as a pivotal player in the development of diabetes (Burcelin, 2016; Tesch, 2017; Xu and Chen, 2017). Here, spontaneous diabetic Lepr^db/db^ and heterozygous Lepr^db/m^ mice were used to evaluate the contribution of the intestinal flora to the progression of inflammation, particularly in the context of obesity-driven retinopathy and nephropathy.

## Materials and methods

### Animals and microbiota transplantation

Male B6.BKS (D)-Lepr^db^/J (stock number: 000697) homozygous Lepr^db/db^ mice (*n* = 16) were used as diabetic mice, and heterozygous Lepr^db/m^ mice (*n* = 16) were used as controls (denoted as db/db and db/m, respectively, hereafter). All mice were purchased from The Model Animal Research Center of Nanjing University (Nanjing, Jiangsu, China) and housed in the Center for New Drug Safety Evaluation of Lunan Pharmaceutical (Lunan Pharmaceutical Group Co., Ltd., Linyi, China) in a pathogen-free, temperature-controlled environment with a 12-h light/dark cycle. The animals were supplied with a regular-chow diet (RD) (16% kcal fat, 63% kcal carbohydrate, 21% kcal protein) and water *ad libitum*. Mice were housed in groups with five mice per cage under SPF conditions with corncob bedding. Throughout the experiment, animal welfare was ensured. All experiments were performed in accordance with the experimental protocols previously approved by the institution’s Animal Ethics Committee at the Pharmacological Center of Lunan Pharmaceutical Group Co., Ltd. and were performed in accordance with the US National Institutes of Health Guidelines for the Care and Use of Laboratory Animals [National Research Council (US) Committee for the Update of the Guide for the Care and Use of Laboratory Animals]. Humane endpoints were implemented according to Ashall and Millar (2014).

At the age of 12 weeks, Lepr^db/db^ (*n* = 8) and Lepr^db/m^ mice (*n* = 8) received antibiotic treatment with 0.5 g/L neomycin trisulfate salt hydrate in their drinking water. Another 8 mice of both the Lepr^db/db^ and Lepr^db/m^ lines were treated with control water. All mice were housed for another 24 weeks, and fecal pellets and blood were collected before sedation with isoflurane gas and cervical dislocation. The eyes and kidneys were fixed in 4% paraformaldehyde buffer for 24 h and embedded in paraffin for further assays.

For microbiota transplantation, 16 6-week-old mice (Lepr^db/db^) started on a regular diet for 1 week and subsequently underwent gut microbiota depletion with antibiotics in their drinking water (ampicillin 1.0 g/L + neomycin 0.5 g/L) for 5 days as described previously (Ellekilde et al., 2014). Two days after discontinuing the antibiotics, 8 mice received their first microbiota transplant from donor mice (Lepr^db/m^) on a RD, and another 8 mice received PBS treatment.

Fecal pallets were collected from 10 different donor mice on RD treatment in 2 ml of PBS (sterile, with 0.05% cysteine HCl). After homogenization and centrifugation (200 g, 5 min), the supernatant (200 μl) was administered orally to the mice with gavage needles. The fresh fecal samples were repeated every 7 days for the duration of the experiment (until the age of 36 weeks). The mice were weighed weekly, and feces were collected for analysis by 16S sequencing.

### Cytokine assessment

For serum cytokine detection, flow cytometry and enzyme-linked immunosorbent assay (ELISA) were performed as described previously (Li et al., 2019). For flow cytometry detection, mouse spleen tissues were minced into 5 ml of PBS buffer containing 1% FBS and then gently mashed through a 200 mesh nylon membrane filter. Splenocytes were collected by centrifugation at 250 × g and 4°C for 10 min. The red blood cells were removed using lysis buffer (KHCO_3_ 0.01 M, NH_4_Cl 0.15 M, and EDTA-Na_2_ 0.1 M, pH 7.4) for 1 min at 37°C. The remaining cells were washed with PBS and resuspended in RPMI-1640 medium containing 10% FBS, 100 IU/ml penicillin and 100 IU/ml streptomycin and then counted. Subsequently, 2 × 10^6^ cells per well were cultured in 6-well plates and incubated with phorbolmyristate acetate (PMA, 100 ng/ml), ionomycin (500 ng/ml) and Brefeldin A (BFA, 10 μg/ml) for 6 h at 37°C. Splenocytes were collected and stained with FITC-anti-mouse CD3 and APC/Cy7-anti-mouse CD4 antibodies at 4°C for 30 min. After washing, the cells were fixed and permeabilized using a Fix&Perm kit (BD Biosciences) and then divided into two equal parts. One part was incubated with APC-anti-TNF-α, PE-anti-IFN-γ, and PE-anti-IL-4 (Biolegend) antibodies, and the other part was incubated with APC-anti-IL-10 and PE-anti-IL-17antibodies for 60 min at 4°C. The samples were analyzed using flow DxFLEX (Beckman).

### Immunohistochemistry and periodic acid-schiff stain assay

Eyes and kidneys were fixed for 24 h in 4% PFA at room temperature before dissection of the sclera–choroid–RPE cell complex. After secondary fixation for15 min in 4% PFA at room temperature, the choroids were stained with rhodamine-labeled *Griffonia* (*Bandeiraea*) *simplicifolia* Lectin I (Vector Laboratories Inc.) in 1 mM CaCl_2_ in PBS and IBA-1 (rabbit polyclonal; Wako).

The following antibodies were used: rabbit monoclonal specific for VEGFA (ab52917), mouse monoclonal specific for TGF-β1 (ab190503), rabbit monoclonal specific for IL-10 (ab133575), rabbit monoclonal specific for TNF-α (ab215188), mouse monoclonal specific for IFN-γ (ab25014), rabbit polyclonal specific for IL-1β (ab2105), rabbit polyclonal specific for IL-6 (ab7737), and mouse monoclonal specific for IL-17A (ab189377).

### Enzyme-linked immunosorbent assay detection

Briefly, mouse sera were collected from each group and analyzed using ELISA kits from Biolegend. According to the manufacturer’s instructions, precoated 96-well strip microplates for TGF-β1, IL-10, TNF-α, IFN-γ, and IL-6 were washed 4 times using 1 × washing buffer. Then, standard dilutions or serum samples were added and incubated at room temperature for 2 h. After washing, different detection antibodies were added and incubated at room temperature for 1 h, followed by incubation with Avidin-HRP D solution. Then, 100 μl of Substrate Solution D was added to each well and incubated for 20 min in the dark, and Stop Solution was added. Absorbance was read at 450 nm. For IL-1β or IL-17A detection, capture antibodies specific for IL-1β or IL-17A were coated onto ELISA plates at 4°C overnight. The other assay procedures were similar to those described above.

### Intestinal permeability and RAW-blue assays

For the intestinal permeability assay, all mice were administered 1 ml of Evans Blue (50 mg/ml) by oral gavage, and 100 μl of submandibular vein blood was collected after 24 h. Evans Blue concentrations in the serum were detected by spectrophotometry at an optical density of 620–740 nm and quantified with a standard dilution curve.

For the RAW-blue assay, murine RAW 264.7 macrophage-derived RAW-blue™ cells that express many pattern recognition receptors (PRRs), including Toll-like receptors (TLRs), NOD-like receptors (NLRs), RIG-I-like receptors (RLRs) and C-type lectin receptors (CLRs) (InvivoGen, San Diego, CA, USA), were used to detect the activity of pathogen-associated molecular patterns (PAMPs) according to the manufacturer’s protocol. Briefly, after being cultured in growth medium (DMEM containing 4.5 g/L glucose, 2 mM L-glutamine, 10% fetal bovine serum (FBS), and 100 mg/L Zeocin) to passage 15, 10^5^ RAW-blue cells were plated in 96-well plates and starved for 6 h with 30 μl of mouse serum. Subsequently, 30 μl of FBS (as a control) or 30 μl of LPS was added to each well and incubated for 21 h at 37°C. Secreted embryonic alkaline phosphatase (SEAP) was detected by spectrophotometry at 620–655 nm after a 1–3-h incubation at 3°C in 20 μl of induced RAW-blue cell supernatant with 180 μl of QUANTI-Blue (InvivoGen, San Diego, CA, USA).

### Real-time PCR analysis

To evaluate mRNA expression, total RNA was extracted from mouse plasma after different treatments using TRIzol reagent according to the manufacturer’s protocol. cDNA was synthesized using a cDNA Synthesis Kit. All real-time PCRs with SYBR Green were performed and analyzed with an ABI Prism 7,500 instrument (Applied Biosystems, Foster City, CA). β-actin was used for normalization of relative expression levels. The primer sequences of each target are shown in Supplementary Figure 2.

### 16S rRNA gene extraction, amplification and sequencing

The mouse feces were collected, and total DNA was extracted using an E.Z.N.A.^®^ Bacterial DNA Kit (Omega Bio-tek, Norcross, GA, USA) according to the manufacturer’s instructions. The V4 segment of the 16S rRNA gene from a single gut microbiome sample was amplified, and microbiome sequencing was performed by GuheInfo-technology Co., Ltd. (Hangzhou, China) as previously described (Gao et al., 2018). Extraction of DNA from fecal pellets was performed according to the manufacturer’s instructions for the Qiagen QIAamp Fast Stool Mini Kit (Cat No. 19593, Qiagen Inc., Valencia, CA). Briefly, 500 μl of Inhibit EX Buffer was added to 5 fecal pellets from each mouse and homogenized by a disposable homogenizing pestle and vortex. After 5 min of heating at 70°C, the suspension was centrifuged at 20,000 × g for 1 min. The supernatant was thoroughly mixed with 20 μl of proteinase K, the mixture was incubated with 500 μl of Buffer AL at 70°C for 10 min, and the lysate (plus 500 μl of 100% ethanol) was centrifuged at 20 000 × g for 1 min. After 500 μl of buffer AW1 was added to the spin column and centrifuged at 20 000 × g for 1 min, the filtrate was discarded, and this step was repeated with 500 μl of buffer AW2. The spin column was dried by centrifugation for 3 min at 20,000 × g in a clean 2-ml collection tube. The DNA was eluted in 200 μl of Buffer ATE, directly pipetted onto the QIAamp membrane, and collected in a clean Eppendorf tube. After the genomic DNA was normalized to 30 ng per PCR, the V3-V4 dual-index fusion PCR primer cocktail and PCR master mix were added, and then PCR was performed with 30 cycles and a melting temperature of 56°C. Non-specific PCR products were purified with AmpureXP beads and sequenced on an Illumina HiSeq 2,500 platform following the standard I llumina pipelines, generating 2 × 250 bp paired-end reads.

### Statistical analysis

Data are expressed as the mean ± S.D. One-way ANOVA followed by Tukey’s *post hoc* test was used to compare multiple treatment groups. Two-way ANOVA was used to assess the statistical significance of the differences among multiple treatment groups at different time points. Statistical significance was set at *P* < 0.05. The data and statistical analysis complied with the recommendations for experimental design and analysis in pharmacology (Curtis et al., 2018).

## Results

### Obesity modulates metabolic variables, exacerbates retinopathy and nephropathy

**FIGURE 1:**
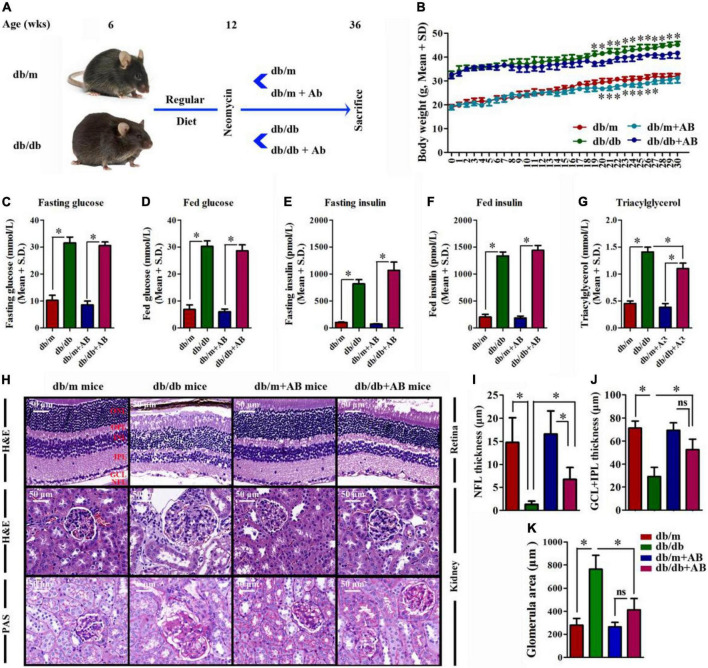
The effect of obesity on metabolic variables exacerbated retinopathy and nephropathy. **(A)** Schematic representation of experimental timeline where half of the mice (Lepr^db/db^ and Lepr^db/m^) start a regular diet (RD) at 6 weeks and later half of these receive neomycin (AB) treatment from the age of 12 weeks until killing at week 36. **(B)** Weight gain of AB-treated mice compared to control mice; Error bars represent mean ± S.D. *n* = 8 in per group. *P < 0.05. **(C–G)** The effect of obesity on metabolic variables including Blood glucose **(C,D**), insulin **(E,F)**, and triacylglycerol **(G)** levels after 16 h of fasting **(C,E)**, and in the random fed state **(D,F)** in mice with or without AB-treatment. *n* = 8 in per group. *P < 0.05. **(H–K)** HE staining was performed to detect vessels morphological changes of retinal sections (**H**, Upper) followed by quantitation of the thickness of NFL **(I)** and GCL + IPL **(J)**. HE (**H**, Middle) and Periodic Acid-Schiff (PAS) (**H**, Bottom) staining was performed to detect glomerulus morphological changes of kidney sections followed by quantitation of the glomerular area **(K)**. Error bars represent mean + S.D. *n* = 8 in per group. *P < 0.05.

In light of epidemiologic data linking obesity to metabolic diseases (Neeland et al., 2018), DR (Sasongko et al., 2018), and DN (Fervenza and Sethi, 2019), we investigated the ability of obesity to influence metabolic variables, retinopathy and nephropathy, and the gut microbiota. Spontaneous diabetic Lepr^db/db^ and heterozygous Lepr^db/m^ mice were raised on a RD (16% kcal fat) from 6 weeks of age and subsequently underwent 24 weeks (from 12 to 36 weeks old) of treatment with 0.5 g/L antibiotic (AB, neomycin trisulfate salt hydrate) in drinking water or normal water (Figure 1A). As expected, upon killing at 36 weeks, Lepr^db/db^ and Lepr^db/db^ + AB mice gained over 48.3 and 47.2% more weight than Lepr^db/m^ and Lepr^db/m^ + AB mice, respectively. Notably, AB treatment did not influence body weight in either Lepr^db/db^ or Lepr^db/m^ mice (Figure 1B). To understand the effect of obesity, metabolic variables including body weight (g), blood glucose (mmol/L), insulin (pmol/L), and triacylglycerol (mmol/L) in this research were measured in Lepr^db/db^ or Lepr^db/m^ mice with or without AB treatment. Fasting and fed blood glucose and insulin levels were significantly higher in Lepr^db/db^ mice than in Lepr^db/m^ mice, even with AB treatment. In addition, the above parameters were not significantly influenced by AB treatment among mice of the same genotype (Figures 1C–F). Interestingly, although triacylglycerol was extremely elevated in Lepr^db/db^ and Lepr^db/db^ + AB mice compared with Lepr^db/m^ and Lepr^db/m^ + AB mice, respectively, AB treatment significantly reduced triacylglycerol in Lepr^db/db^ mice but not in Lepr^db/m^ mice (Figure 1G). To further investigate the pathological influences of obesity on the kidney and retina, H&E and Periodic Acid-Schiff (PAS) staining was performed on sections of kidney and retina at 36 weeks of age. Compared with that in Lepr^db/db^ mice, the thickness of NFL and GCL + IPL in the retina of Lepr^db/m^ mice increased over 10- and 2.5-fold (NFL, 1.33 ± 0.68 μm; GCL + IPL, 29.1 ± 8.15 μm for Lepr^db/db^ vs. NFL, 14.77 ± 5.38 μm; GCL + IPL, 71.2 ± 6.1 μm for Lepr^db/m^), respectively. In contrast, the same severity of diabetes plus AB treatment significantly ameliorated retinopathy (Figure 1H, upper; Figures 1I,J) (6.73 ± 2.60 μm for NFL and 52.57 ± 9.13 μm for GCL + IPL, respectively). For the kidney, H&E and PAS staining showed that compared with that in Lepr^db/m^ mice, the glomerular area was clearly increased in Lepr^db/db^ mice (763.33 ± 12.56 μm^2^ in Lepr^db/db^ vs. 280.02 ± 57.66 μm^2^), and AB inhibited glomerular hypertrophy in Lepr^db/db^ mice (413.34 ± 97.86 μm^2^) (Figure 1H, middle and bottom; Figure 1K). These results showed that obesity not only modulates metabolic variables but also exacerbates retinopathy and nephropathy and that oral neomycin could obviously reduce triacylglycerol levels and prevent obesity-induced pathological changes in the kidney and retina, although neomycin showed no effects on glucose or insulin levels compared with untreated obese mice. As a broad-spectrum, non-gut permeable antibiotic, neomycin was used to tease out the contribution of gut microbes to heightened retinopathy and nephropathy in obesity; the results strengthened the link between the gut flora and pathological angiogenesis.

### Obesity-induced gut microbiota dysbiosis correlates with the severity of retinopathy and nephropathy

**FIGURE 2:**
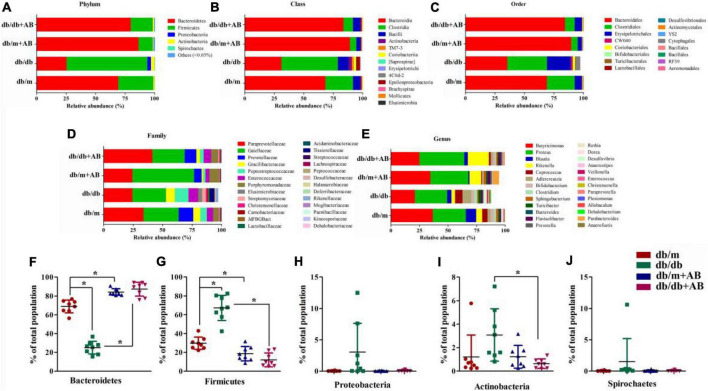
Obesity induced gut microbiota dysbiosis. Representative charts of relative abundance of bacterial phyla **(A)**, class **(B)**, order **(C)**, family **(D)** and genus **(E)** in gut microbiota of Lepr^db/db^ and Lepr^db/m^ mice treated with neomycin or not, and relative proportion per group of different phyla **(F–J)**. *n* = 8 in per group. *P < 0.05.

To investigate whether obesity and oral neomycin modify the intestinal flora, we next profiled the gut microbiome. We characterized gut microbiome composition by sequencing the V3-V4 hypervariable regions of bacterial 16S rRNA extracted from fecal pellets originating from Lepr^db/db^ and Lepr^db/m^ mice that did or did not receive neomycin^1^ (Figures 2A–E and Supplementary Table 1). Previous studies demonstrated that as two dominant phyla, the ratio of *Bacteroidetes* to *Firmicutes* accounts for over 90% of bacterial phylogenetic types in the distal gut (Arumugam et al., 2011; Lozupone et al., 2012). The sequencing quality was evaluated by rarefaction analysis methods based on alpha diversity indexes, as shown in Supplementary Figure 1 and Supplementary Table 2. The results showed that compared with Lepr^db/m^ mice, Lepr^db/db^ mice had shifted ratios of commensal gut microbes; for example, the Bacteroidetes/Firmicutes ratios shifted from 68.9 to 29.8% of total bacteria in Lepr^db/m^ mice to 25.1 to 67.3% in Lepr^db/db^ mice. Importantly, oral neomycin elevated the proportion of *Bacteroidetes* (up to 87.2% in Lepr^db/db^ + AB and 84.2% in Lepr^db/m^ + AB, respectively) among total bacteria and reduced the proportion of *Firmicutes* (to 12.0% in Lepr^db/db^ + AB and 18.6% in Lepr^db/m^ + AB, respectively) (Figures 2F,G). In addition, except for *Bacteroidetes* and *Firmicutes*, there are three other phyla with the highest content (*Proteobacteria*, *Actinobacteria*, and *Spirochetes*) that were calculated simultaneously. As a microbial signature of dysbiosis of the gut microbiota, the relative abundance of *Proteobacteria* rose with obesity (from 0.05% in Lepr^db/m^ to 3.04% in Lepr^db/db^) and was then reduced to 0.08% after antibiotic treatment (Figure 2H) Notably, Lepr^db/db^ mice host the most diverse microbiome, with a modest but important presence of *Actinobacteria* and *Spirochetes* (3.08 and 1.51% of the total) (Figures 2I,J). These phyla were inhibited in Lepr^db/m^ mice or after receiving oral neomycin. Together, these data show that modulation of the gut microbiota correlates with the severity of retinopathy and nephropathy.

### Obesity augments gut permeability, metabolic endotoxemia, and systemic inflammation

In obesity, the altered microbiota in the gut has been demonstrated to disturb the barrier function of the gut epithelial layer, resulting in increased PAMPs in the systemic circulation (Kamada et al., 2013; Kuntzen and Schwabe, 2017; van den Munckhof et al., 2018). PAMPs are recognized by PRRs at low concentrations under physiologic conditions (DiBaise et al., 2012), but this effect increases in the presence of LPS, which subsequently provokes an inflammatory reaction (Kearney and Martin, 2017), insulin resistance (Clark and Vissel, 2018), fatty liver disease (Krenkel and Tacke, 2017), retinopathy (Wang et al., 2015) and nephropathy (Wada and Makino, 2016).

**FIGURE 3:**
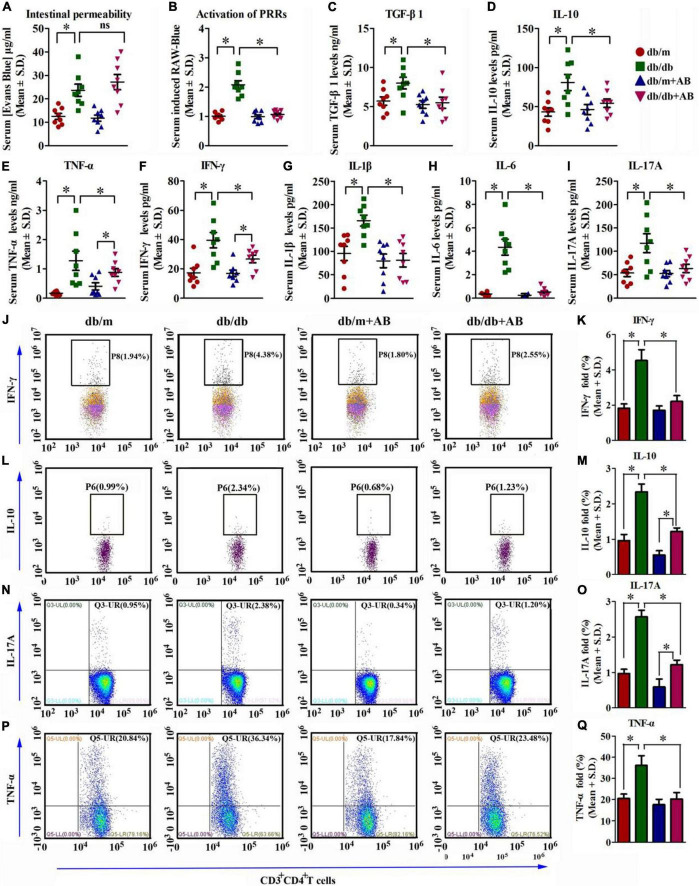
Obesity augments gut permeability, metabolic endotoxemia, and systemic inflammation. **(A)** Concentration of gut-absorbed Evans Blue in serum 24 h after oral administration in db/db and db/m mice, treated with vehicle or with antibiotic. *n* = 8 in per group. *P < 0.05. **(B)** Activation of PRRs induced by stimulation with serum isolated from db/db and db/m mice and receiving vehicle or neomycin for 3 weeks. *P < 0.05. **(C–I)** Serum cytokine profiles determined by ELISA assay of TGF-β1 **(C)**, IL-10 **(D)**, TNF-α **(E)**, IFN-γ **(F)**, IL-1β **(G)**, IL-6 **(H)**, and IL-17A **(I)** in db/db and db/m mice, treated with vehicle or with antibiotic. *n* = 8 in per group. *P < 0.05. **(J–Q)** IFN-γ **(J,K)**, IL-10 **(L,M)**, IL-17A **(N,O)**, and TNF-α **(P,Q)** levels were assayed by flow cytometry. *n* = 8 in per group. *P < 0.05.

To analyze whether intestinal permeability was influenced by obesity and/or antibiotics, the Evans Blue assay was performed, and the results showed that obesity enhanced the intestinal permeability by 3.2-fold compared with that of the control. In contrast, antibiotic treatment slightly but did not significantly restore intestinal permeability in either Lepr^db/db^ or Lepr^db/m^ mice (Figure 3A).

Subsequently, whether PRR responses were triggered by PAMP levels in the circulation of Lepr^db/db^ and Lepr^db/m^ mice with or without antibiotic treatment was detected in RAW-blue cells. The RAW-blue cells were incubated with mouse serum from different treatment groups to detect the activation of PRRs due to the cells containing chromosomal integration of a SEAP reporter, which is inducible by NF-κB and AP-1 and expresses many PRRs (such as TLRs, NLRs, RLRs, and CLRs). The results showed that serum from Lepr^db/db^ mice activated PRRs significantly compared with serum from either Lepr^db/m^ mice or Lepr^db/db^ mice receiving antibiotic treatment (Figure 3B).

We next investigated systemic profiles of classical inflammatory factors in mice under various paradigms. Consistent with the heightened activation of PRRs, ELISA showed that serum cytokine concentrations of TNF-α, IFN-γ, IL-1β, IL-6, and IL-17A were significantly induced with obesity, as was anti-inflammatory TGF-β and IL-10. The oral intake of AB reversed the trend (Figures 3C–I). Moreover, the flow cytometry (FCM) results were in accordance with the ELISA results. As FCM showed, inflammation-related factors including TNF-α, IFN-γ, and IL-17A were all high in db/db mice compared with db/m mice. While db/m and db/db mice were treated with AB, these inflammatory factors all declined, especially in db/db mice; IFN-γ changed from 4.38 to 2.55%, IL-17A changed from 2.38 to 1.20%, and TNF-α changed from 36.34 to 23.48% (Figures 3J–Q). These results suggest that neomycin trisulfate salt hydrate could efficiently improve systemic inflammation in db/db mice. In addition, this pattern was confirmed by investigating the transcript levels of the above pro- and anti-inflammatory factors as well as the cardinal angiogenic factor *vegfa* (Supplementary Figure 2).

### Obesity promoted local inflammation in retina and kidney

**FIGURE 4:**
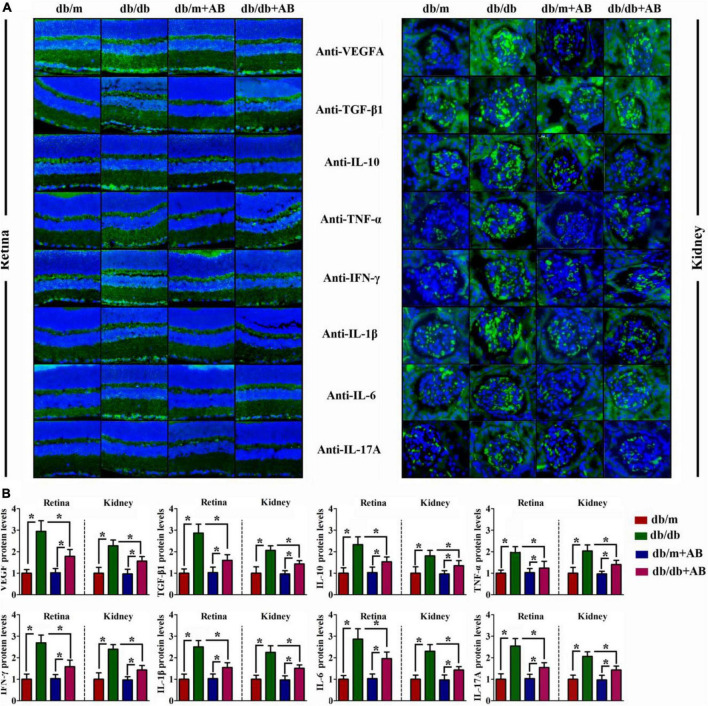
Obesity promoted local inflammation in retina and kidney. Representative immunofluorescence images of retina (**A**, Left) and kidney (**A**, Right) tissues stained with VEGFA, TGF-β1, IL-10, TNF-α, IFN-γ, IL-1β, IL-6, and IL-17A antibodies in db/db and db/m mice, which were treated with vehicle or with antibiotic. The relative quantitative analyses are presented in **(B)**. Bar = 50 μm. Data are expressed as the means ± S.D., *n* = 8 in per group. *P < 0.05.

Immunohistochemical staining was performed to further investigate the change above in serum cytokine concentrations and anti-inflammatory TGF-β and IL-10 after retention in the retina (Figure 4A, left) and kidney (Figure 4A, right). As suspected, compared with Lepr^db/m^ mice, expression of not only proinflammatory factors such as TNF-α, IFN-γ, IL-1β, IL-6, and IL-17A but also anti-inflammatory factors such as TGF-β and IL-10 was enhanced in both retained and kidney tissues of Lepr^db/db^ mice; similar results were obtained for VEGFA. In addition, neomycin treatment of Lepr^db/db^ mice improved their expression (Figures 4A,B). These data suggest that obesity increased intestinal permeability, metabolic endotoxemia, and systemic inflammation in a gut flora-dependent manner, with gut microbial community dysbiosis resulting in alterations of circulating levels of pro- and anti-inflammatory cytokines and the expression of inflammation-associated mRNAs.

### Microbiota transplants confirm that obesity aggravates retinopathy and nephropathy through the gut microbiota

**FIGURE 5:**
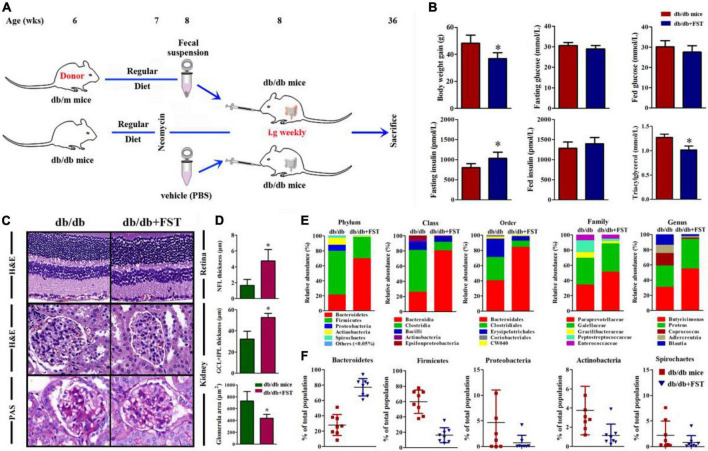
Obesity exacerbated DR, DN, and inflammaging condition through gut microbiota. **(A)** Schematic representation of microbiotal transfer experiments where recipient mice (db/db) were gavaged with a suspension of fecal pellets in PBS from donor mice (db/m) or vehicle. The experimental timeline describing preparation of mice for microbiotal transfers where mice received 7 days of antibiotics (neomycin and ampicillin) at 6 weeks of age. Starting at 8 weeks of life, db/db mice received weekly microbiotal transplants from db/m donor mice until killing at week 36. **(B)** Body weight, blood glucose, insulin, and triacylglycerol levels after 16 h of fasting, and in the random fed state in mice with or without microbiotal transfers. *n* = 8 in per group. *P < 0.05. **(C)** HE staining was performed to detect vessels morphological changes of retinal sections (**C**, Upper), HE (**C**, Middle) and PAS (**C**, Bottom) staining was performed to detect glomerulus morphological changes of kidney sections. **(D)** Quantitation of the thickness of NFL(**D**, Upper), GCL + IPL (**D**, Middle) and the glomerular area (**D**, Bottom) in db/db mice with or without microbiotal transfers were followed. Error bars represent mean + S.D. *n* = 8 in per group. *P < 0.05. **(E,F)** Representative charts of relative abundance of bacterial phyla, class, order, family **(E)** and genus in gut microbiota of db/db mice with or without microbiotal transfers, and relative proportion per group of different phyla **(F)**. *n* = 8 in per group. *P < 0.05.

To confirm whether obesity-induced gut microbiota dysbiosis is associated with retinopathy and nephropathy, fecal microbiota harvested from the feces of Lepr^db/m^ mice were transplanted by oral gavage to Lepr^db/db^ mice (db/db + FST), and Lepr^db/db^ mice transplanted with PBS served as controls. Before microbiota transplantation, Lepr^db/db^ (recipient) mice were treated with ampicillin and neomycin in their drinking water to deplete their original commensal microbiome, and gavage with FST was performed once a week to maintain a constant composition of the transplanted flora (Figure 5A). Flora transplantation significantly altered body weight, serum insulin (fasting) and triacylglycerol levels compared with those of PBS-treated mice. In contrast, serum glucose (under both fasting and fed conditions) and fed insulin levels were not influenced by flora transplantation (Figure 5B).

**FIGURE 6:**
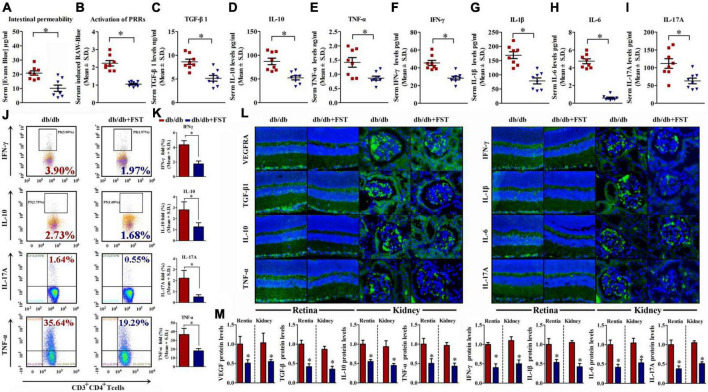
Microbiotal transfers ameliorated gut permeability, metabolic endotoxemia, and inflammaging condition in obesity mice. **(A,B)** Concentration of gut-absorbed Evans Blue **(A)** in serum 24 h after oral administration and activation of PRRs **(B)** of db/db mice with or without microbiotal transfers. *n* = 8 in per group. *P < 0.05. **(C–I)** Serum cytokine profiles of db/db mice with or without microbiotal transfers determined by ELISA assay of TGF-β1 **(C)**, IL-10 **(D)**, TNF-α **(E)**, IFN-γ **(F)**, IL-1β **(G)**, IL-6 **(H)**, and IL-17A **(I)**. *n* = 8 in per group. *P < 0.05. **(J,K)** IFN-γ, IL-10, IL-17A, and TNF-α levels were assayed by flow cytometry. *n* = 8 in per group. *P < 0.05. **(L,M)** Representative immunofluorescence images of retina and kidney tissues stained with VEGFA, TGF-β1, IL-10, TNF-α, IFN-γ, IL-1β, IL-6, and IL-17A antibodies in db/db mice with or without microbiotal transfers **(L)**. The relative quantitative analyses were presented in **(M)**. Bar = 50 μm. Data are expressed as the means ± S.D., *n* = 8 in per group. *P < 0.05.

According to the above results, obesity exacerbates retinopathy and nephropathy. FST-treated Lepr^db/db^ simultaneously ameliorated retinopathy and nephropathy by increasing the thickness of NFL and GCL + IPL (4.87 ± 1.79 μm for NFL and 54.21 ± 5.14 μm for GCL + IPL, respectively) and diminishing the glomerular area (∼42%) (Figures 5C,D). To verify successful transplantation of diet-associated microbial phyla, we characterized the fecal microbiota as discussed above, and the results demonstrated that in PBS-treated Lepr^db/db^ mice, *Firmicutes* was the primary phylum and accounted for 38–79% of the microbiome, but this value diminished to 3–35% after FST-treatment mice. In contrast, increase of *Bacteroidetes* in Lepr^db/db^ mice was observed after fecal transplantation (from 5–58 to 60–92%). Not only *Firmicutes* and *Bacteroidetes* but also other phyla, including *Proteobacteria*, *Actinobacteria*, and *Spirochetes*, were restored in FST-mice (Figures 5E,F). In addition, our data showed that intestinal permeability was decreased in Lepr^db/db^ mice with microbiota transfer compared to PBS transfer control (Figure 6A). The activation of PRRs was inhibited by serum from FST-mice compared with PBS-treated mice (Figure 6B). Subsequent assays, such as ELISA (detecting serum proinflammatory factors TNF-α, IFN-γ, IL-1β, IL-6, IL-17A, and anti-inflammatory factors TGF-β and IL-10) (Figures 6C–I), flow cytometry (TNF-α, IFN-γ, IL-17A, and IL-10) (Figures 6J,K), immunohistochemistry in the retina and kidney (Figures 6L,M), and QRT-PCR (Supplementary Figure 2) confirmed that the transfer of fecal suspensions to Lepr^db/db^ mice reduced overall inflammation. In concert, these results provide evidence for the influence of gut microbiota on retinopathy and nephropathy.

## Discussion

With an increasing prevalence of obesity, the incidence of obesity-induced diabetes and diabetic complications is booming, consequently dramatically increasing societal impacts and the financial burden in the coming years (Lavin et al., 2016; Pollack et al., 2016). Current medical approaches cannot meet people’s life expectancy demands, and we urgently need minimally intrusive and cost-effective paradigms to prevent or delay diabetic complications, such as DR and DN (Barchetta et al., 2016). As diabetes-induced microvascular diseases, DR and DN are considered the most common complications of diabetes and are now well established as the association between inflammation and microvascular disease (Diabetes Prevention Program Research Group, 2015; Hadjadj et al., 2016; Sohn et al., 2016). The most widely utilized animal model of diabetes is Lepr^db/db^ mice, which have a leptin receptor mutation and represent an excellent animal model of diabetic complications (Forbes and Cooper, 2013). In this study, Lepr^db/db^ and control mice (Lepr^db/m^) were fed a regular diet and housed for 36 weeks of age. The metabolic variables and pathological examination demonstrated that Lepr^db/db^ mice significantly enhanced body weight (approximately 1.8-fold vs. Lepr^db/m^ mice), elevated glucose, insulin, and triacylglycerol, and exacerbated retinopathy and nephropathy compared with Lepr^db/m^ mice.

**FIGURE 7:**
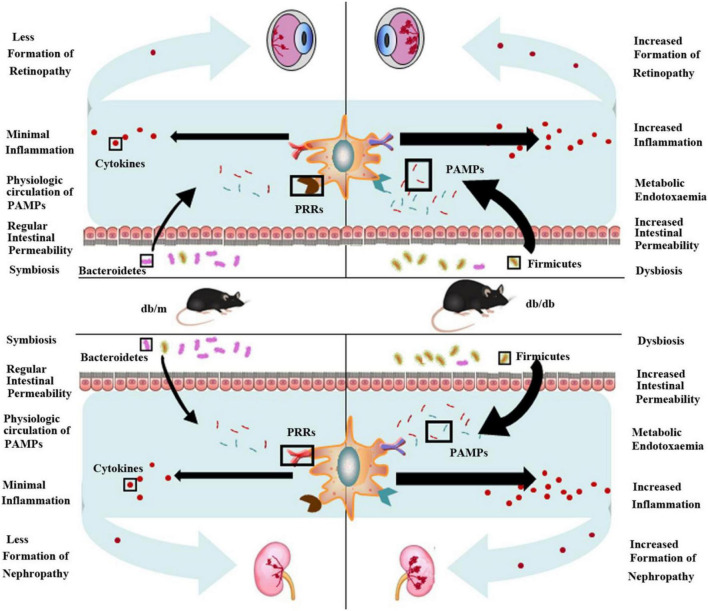
Obesity-induced gut microbiota dysbiosis increases intestinal permeability, metabolic endotoxemia, and systemic and local inflammation and ultimately contributes to retinopathy and nephropathy. The ratio of two dominant phyla in intestinal flora, Bacteroidetes and Firmicutes significantly decreases in obesity (db/db) mice compared to normal (db/m) mice, with a relative increase in Firmicutes in obesity mice and decrease in Bacteroidetes. This dysbiosis is accompanied by heightened intestinal permeability, which allows increased translocation of pathogen-associated molecular patterns (PAMPs) (endotoxemia). Recognition of these microbe-specific molecules by pattern recognition receptors (PRRs) present on innate immune cells triggers synthesis and excretion of cytokines causing chronic systemic low-grade inflammation. Ultimately, this exacerbates retinopathy and nephropathy.

Growing evidence suggests that long-term chronic inflammation induced by the innate immune system activation elicited disease instead of repair, leading to the development of DR and DN (Borilova Linhartova et al., 2016; Volpe et al., 2018). In addition, it has long been known that the gut microbiota, a population consisting of an astonishing number of members, is present in the human intestine; these organisms were long neglected but subsequently proved to be essential for human health (Lynch and Pedersen, 2016; Marchesi et al., 2016). After a billion years, the coevolution of mammals and the microbiota finally led to interdependency and intricate interactions among microbiota, immunity, and human health/disease (Lynch and Pedersen, 2016). Our study suggests that the gut microbiota influences the development of retinopathy and nephropathy, particularly when obesity is a predisposing factor (Figure 7). We show that the gut microbiome was altered in Lepr^db/db^ mice and in turn elevated systemic inflammation and ultimately increased pathological features of retina and kidney tissue. This effect could originate from increased intestinal permeability to PAMPs secondary to dysbiosis. In this regard, previous research has found that gut microbiota dysbiosis is associated with obesity and can attenuate gut barrier function, which increases the entry of PAMPs into the systemic circulation and consequently triggers inflammation (Osborn and Olefsky, 2012). We found that two dominant phyla, *Bacteroidetes* was decreased and *Firmicutes* was increased in Lepr^db/db^ mice, which was similar to that in the earlier studies (Sedighi et al., 2017; Zhao et al., 2019). *Bacteroidetes* is reported to improve glucose metabolism disorders and restore insulin sensitivity and profit maintaining the integrity of the intestinal barrier (Xie et al., 2021). *Bacteroidetes* exhibits a negative correlation with fasting glucose levels while *Firmicutes* and *Actinobacteria* are positively correlated with fasting glucose levels (Ahmad et al., 2019). And *Firmicutes* is well known for fat digestion and are associated with obesity (Ahmad et al., 2019). All the results suggest that the imbalance of *Bacteroidetes* and *Firmicutes* correlates with the severity of retinopathy and nephropathy. Therefore, DR or nephropathy could be improved by shifting microbiota composition. And modulation of gut microbiota has been a novel method to ameliorate T2DM. Dietary interventions such as increasing plant-based fibers intake have been identified to be effective in improving microbiota composition (Xiao et al., 2014). And dietary fiber can protect against DN through enrichment of short chain fatty acids (SCFA)-producing bacteria (Li et al., 2020). Probiotics and prebiotics are also commonly used to regulate the gut flora, and the application of synbiotics and probiotics has been found to regulate the metabolic profile of people with diabetes. Synbiotics and probiotics can effectively decrease the inflammatory factors and oxidative stress, which could ameliorate kidney injury in diabetes (Panwar et al., 2013; Zheng et al., 2019). It is reported that supplementation of *Akkermansia muciniphila* in humans improved insulin sensitivity and inflammation, which is safe and has therapeutic potential (Depommier et al., 2019). For DR, modulation of the gut microbiota profile via administration of probiotics has shown positive effects in preclinical mice models (Li et al., 2018), and there are still no studies modulating the microbiome on DR in humans (Iatcu et al., 2021). So further research related to modulation of the gut microbiota involving human trials should be carried out in the future.

PAMPs contribute to the development of inflammation and insulin resistance. LPS as a typical PAMP mainly from the cell wall of Gram-negative bacteria is found to be elevated in the circulation of people with diabetes and increases increased intestinal permeability through the activation of TLRs, mainly TLR4 (Gomes et al., 2017). Activation of TLR pathway induces renal injury and inhibition the pathway by specific antagonists or antibodies reduces proinflammatory cytokine synthesis and oxidative stress in db/db mice (Rayego-Mateos et al., 2020). However, there are no clinical studies targeting TLRs signaling in diabetic kidney disease now. Our study also shows that PRRs activity is enhanced in Lepr^db/db^ mice serum, suggesting that PAMPs levels in the circulation of Lepr^db/db^ is increased. Besides of LPS, there are other PAMPs including flagellin and peptidoglycan, which both are involved in the pathogenesis of inflammation of obesity (Carrizales-Sanchez et al., 2021). But there are few reports on the relationships between the two PAMPs and DN or DR.

To confirm that the gut microbiota plays a pivotal role in modulating intestinal permeability, subsequently adjusting circulatory inflammation and regulating the pathogenesis of disease, Lepr^db/db^ mice underwent antibiotic treatment and microbiota transplants. The results showed that both antibiotic treatment and microbiota transplants from Lepr^db/m^ mice diminished systemic and tissue inflammation, and microbiota transplants significantly lowered intestinal permeability. Compared with antibiotic-treated Lepr^db/db^ mice, the microbial community of mice that received microbiota transplants resembles that of the Lepr^db/m^ mice more closely, which indicates that the effects of neomycin treatment may decrease the absolute number of bacteria present in the gut. Modifying the microbiota can reduce systemic and local inflammation and attenuate pathological processes. Notably, proinflammatory factors such as TNF-α, IFN-γ, IL-1β, IL-6, and IL-17A were significantly elevated in Lepr^db/db^ mice compared with Lepr^db/m^ mice; after altering microbiota constituents via antibiotics or microbiota transplants, as anti-inflammatory factors, TGFβ and IL-10 diminished inflammation in both the circulation (ELISA and QRT-PCR assays were performed to detect inflammatory factor levels in the plasma) and tissues (immunohistochemical assays were performed to detect inflammatory factor levels in tissues). It has been reported that hyperglycemia increases advanced glycationend product formation and reactive oxygen species (ROS) and activates NF-κB, followed by an increase in cytokines (IL-1, IL-6, TNF-α) (Agrawal and Kant, 2014). Our results are consistent with this report. Therefore, targeting inflammatory mediators becomes a potential therapeutic treatment in DR and DN. In addition to the gut microbiome, local intestinal inflammation also influences systematic inflammation. Many developmental aspects of the adaptive immune system are affected by bacterial colonization of the gut. It has been demonstrated that changes in the symbiotic microbiota early in life lead to exacerbated type 2 immunity and allergies. Such changes can also influence Th17 and Treg production in the intestine (Thomas et al., 2017). Therefore, gut microbial dysbiosis is linked to several autoimmune and immune-mediated diseases. Production of some specific cytokines (such as TNFα and IFN-γ) is associated with specific microbial metabolic pathways (Schirmer et al., 2016). Here, we further elaborate the relationship of the gut microbiome with DN and DR, which provides new treatments for DN and DR.

Elevated levels of inflammatory factors are associated with early and late diabetic complications, including DR and DN (Gnudi et al., 2016; Papatheodorou et al., 2016; Napoli et al., 2017), and are significantly related to smoking, drinking, HFD, lack of exercise, and “inflammaging” (Franceschi and Campisi, 2014). Although gut flora dysbiosis may be an additional or minor factor that accounts for the inconsistent responses between individuals subjected to dietary interventions designed to stall the progression of diabetic complications (Walker et al., 2011; Henao-Mejia et al., 2012), histopathological examination provides solid evidence in our research that the reversal of gut flora dysbiosis through antibiotics or microbiota transplants markedly improved the pathological changes of the retina and kidney caused by obesity. From another perspective, the current research strengthens the notion that when designing animal studies, housing and dietary conditions must be taken into account. The gut microbiota are acquired from the surrounding environment shortly after birth and form a relatively stable community, and environmental factors such as diet, exercise, and medication can shift the composition of the microbiota (David et al., 2014; Evans et al., 2014). These findings have broad and significant implications for understanding the role of the gut microbiota in both disease; thus, modifying the gut microbiome may provide minimally intrusive and cost-effective paradigms to prevent or delay exudative retinopathy and nephropathy.

## Data availability statement

The datasets presented in this study can be found in online repositories. The names of the repository/repositories and accession number(s) can be found below: NCBI—PRJNA886966.

## Ethics statement

The animal study was reviewed and approved by the Institution’s Animal Ethics Committee at the Pharmacological Center of Lunan Pharmaceutical Group Co., Ltd.

## Author contributions

Y-SR conceived and designed the study. JL, J-LL, and X-YC conducted the experiments. H-PZ, Y-JT, TC, L-LZ, and ZL interpreted and analyzed the data. JL and Y-SR prepared the draft manuscript. JL and Y-SR reviewed the manuscript. All authors have read and agreed with the published version of the manuscript.

## Footnotes

1
http://www.ncbi.nlm.nih.gov/bioproject/886966

## Supplementary material

Supplementary Figure 1 Rarefaction analysis curve based on alpha diversity. **(A)** Chao index rarefaction curve; **(B)** Shannon index rarefaction curve; **(C)** Simpson index rarefaction curve.

Supplementary Figure 2 mRNA expressions of *vegfa*, *tgf-*β*1*, *il-10*, *tnf-*α, *ifn-*γ, *il-1*β, *il-6*, and *il-17a* in db/db and db/m mice, treated with vehicle or with antibiotic **(A)** and in db/db mice with or without microbiotal transfers **(B)**. Data are expressed as the means ± S.D., *n* = 8 in per group. **P* < 0.05.
