# Supplementary figures and images for: Gut microbiota dysbiosis as an inflammaging condition that regulates obesity-related retinopathy and nephropathy
Source: Front Microbiol. 2022 Nov 2;13:1040846. doi: 10.3389/fmicb.2022.1040846 (PMC9666733; doi:10.3389/fmicb.2022.1040846)

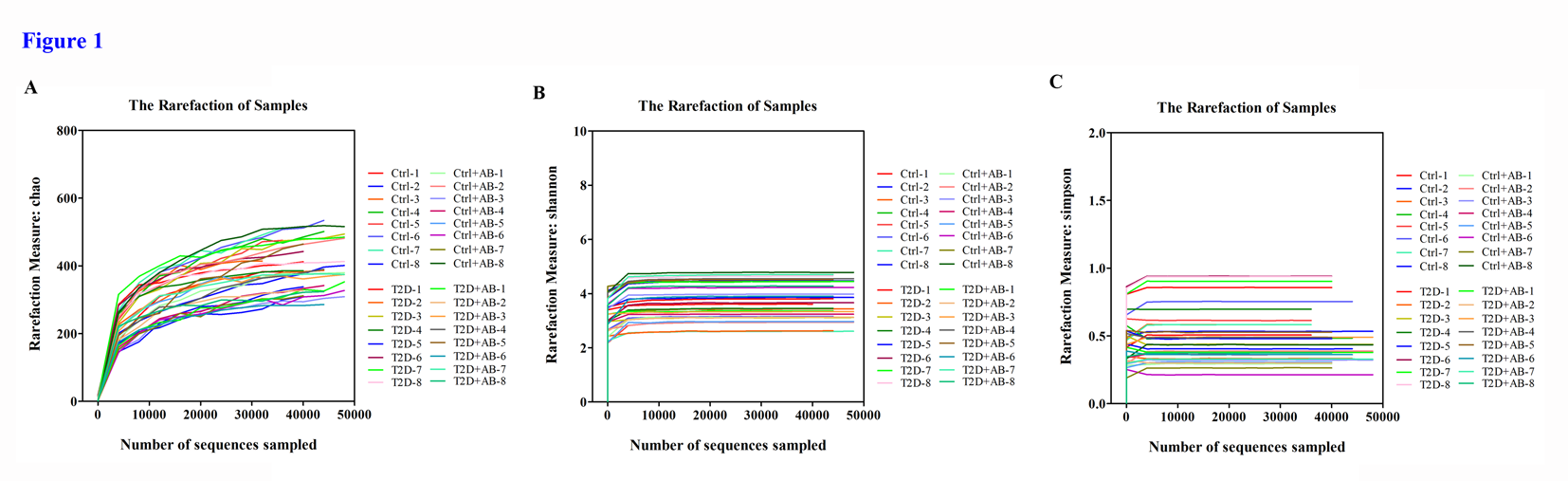

Supplement: Supplementary Figure 1 — Rarefaction analysis curve based on alpha diversity. (A) Chao index rarefaction curve; (B) Shannon index rarefaction curve; (C) Simpson index rarefaction curve. [file Image_1.TIF]

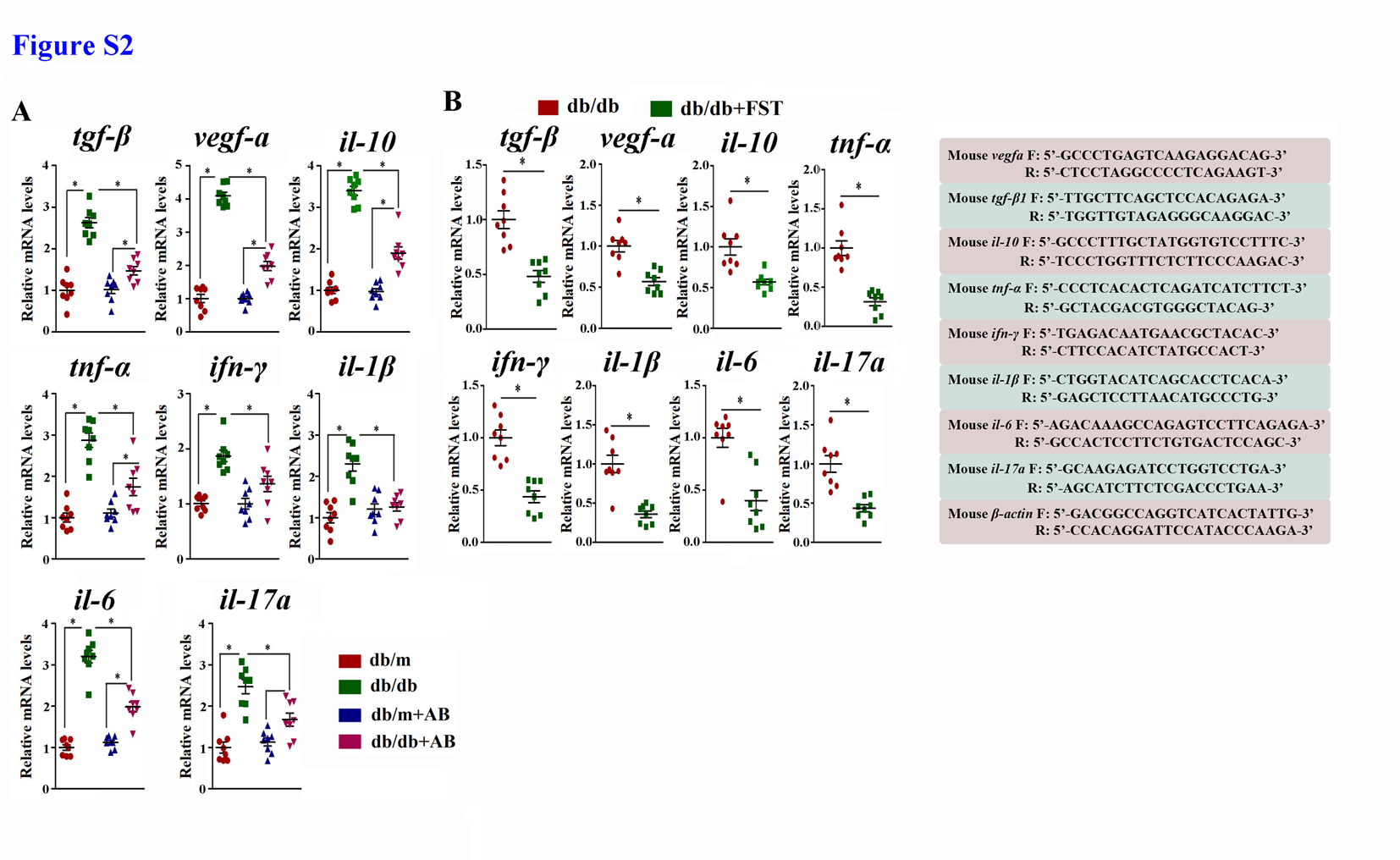

Supplement: Supplementary Figure 2 — mRNA expressions of vegfa, tgf-β1, il-10, tnf-α, ifn-γ, il-1β, il-6, and il-17a in db/db and db/m mice, treated with vehicle or with antibiotic (A) and in db/db mice with or without microbiotal transfers (B). Data are expressed as the means ± S.D., n = 8 in per group. *P < 0.05. [file Image_2.TIF]
